# Code-Free Classification of Pediatric Pneumonia on Chest Radiographs Using Google Cloud Vertex AI AutoML: A Proof-of-Concept Internal Validation Study

**DOI:** 10.7759/cureus.114986

**Published:** 2026-08-22

**Authors:** Mark A Bachir, Alexander S Bachir, Akshay J Reddy, Jasmeen Cheema, Jacob Webster, Telak Brahmbhatt, Rakesh Patel

**Affiliations:** 1 Internal Medicine, California Northstate University College of Medicine, Elk Grove, USA; 2 Medicine, California Health Sciences University, Fresno, USA; 3 Medicine, California University of Science and Medicine, Colton, USA; 4 Medicine, University of California, Davis, Davis, USA; 5 Medicine, The University of Oklahoma, Norman, USA; 6 Medicine, Lincoln Memorial University DeBusk College of Osteopathic Medicine, Knoxville, USA; 7 Internal Medicine, East Tennessee State University Quillen College of Medicine, Johnson City, USA

**Keywords:** artificial intelligence, automated machine learning, chest radiography, cloud-based modeling, computer-aided diagnosis, deep learning, google vertex ai, image classification, medical imaging, pediatric pneumonia

## Abstract

Pneumonia is a clinically significant respiratory infection for which timely and accurate diagnosis is essential. Chest radiography is commonly used in the evaluation of suspected pneumonia; however, radiographic interpretation may be affected by reader experience, workload, image quality, and interobserver variability. This study evaluated whether a commercially available, code-free automated machine learning platform could distinguish pediatric chest radiographs labeled as normal from those labeled as pneumonia.

A publicly available pediatric chest radiograph dataset was uploaded to Google Cloud Vertex AI AutoML Image Classification. Of 5,863 source images, 5,824 were successfully imported, including 1,579 normal radiographs and 4,245 pneumonia radiographs. Vertex AI randomly divided the imported images into training, validation, and testing subsets using an 80-10-10 allocation. A single-label binary image-classification model was trained using an eight node-hour budget, with all remaining user-configurable settings left at platform defaults.

The model achieved an average precision of 0.999. At a confidence threshold of 0.50, platform-reported aggregate precision and recall were both 98.6%. The row-normalized confusion matrix demonstrated correct classification of 97% of normal radiographs and 99% of pneumonia radiographs. Model training was completed in two hours and eight minutes without custom neural-network programming or dedicated local high-performance computing hardware.

These findings demonstrate the technical feasibility of developing a high-performing medical image-classification model using a code-free, cloud-based AutoML platform. However, the results represent internal benchmark performance on a single curated pediatric dataset. Image-level randomization, unavailable patient identifiers, class imbalance, proprietary model development, and the absence of external validation limit clinical generalizability. Independent multi-institutional evaluation is required before clinical implementation can be considered.

## Introduction

Pneumonia is an acute infection of the lung parenchyma and remains an important cause of morbidity, hospitalization, and mortality worldwide, particularly among young children, older adults, and immunocompromised individuals. Timely recognition is essential because delayed diagnosis may contribute to progressive respiratory compromise, prolonged hospitalization, and increased healthcare utilization. Pediatric community-acquired pneumonia guidelines emphasize that diagnosis is primarily clinical and do not recommend routine chest radiography for children who are well enough for outpatient management; chest radiographs are recommended when hypoxemia, significant respiratory distress, failure of initial therapy, hospitalization, or concern for complications is present [1].

Chest radiography remains the primary initial imaging modality for the evaluation of suspected pneumonia because it is widely available, relatively inexpensive, rapidly obtainable, and associated with substantially less radiation exposure than computed tomography [1]. Radiographic manifestations may include focal airspace opacities, multifocal infiltrates, interstitial abnormalities, or lobar consolidation. Interpretation may nevertheless be challenging because these findings can overlap with atelectasis, pulmonary edema, aspiration, viral infection, and other pulmonary conditions. Image quality, reader experience, clinical workload, and interobserver variability may further affect diagnostic performance [2,3].

Artificial intelligence has emerged as a promising approach for assisting with medical image interpretation. Deep-learning techniques, particularly convolutional neural networks, can learn hierarchical imaging features directly from pixel data without requiring manual feature engineering. These methods have demonstrated strong performance across several thoracic imaging tasks, including automated classification of pneumonia on chest radiographs [2,3].

The pediatric chest radiograph dataset introduced by Kermany et al. has become a widely used benchmark for developing and evaluating pneumonia-classification algorithms [4]. Subsequent studies and systematic reviews have reported high diagnostic performance for deep-learning models using this and other chest radiograph datasets [5-7]. Deep-learning systems have also demonstrated performance comparable to practicing clinicians for selected chest radiograph interpretation tasks [8].

Despite these advances, conventional deep-learning model development commonly requires programming expertise, manual architecture selection, hyperparameter optimization, and access to specialized computing hardware [9]. These requirements may limit participation by investigators and institutions without substantial technical resources. Cloud-based automated machine learning platforms address some of these barriers by automating model selection, training, and optimization while providing scalable computational infrastructure [10-13].

However, strong performance on an internal testing dataset does not necessarily indicate that a model will generalize to new hospitals or patient populations. Differences in imaging equipment, acquisition protocols, disease prevalence, patient characteristics, labeling methods, and hidden dataset-specific features may substantially reduce external performance [14]. Transparency and reproducibility may also be limited when proprietary AutoML platforms do not disclose their complete model architectures, preprocessing methods, or optimization procedures [15,16].

The objective of this study was to develop and internally evaluate a binary chest radiograph-classification model using Google Cloud Vertex AI AutoML Image Classification, with pneumonia designated as the positive class. The primary performance endpoint was average precision on the platform-generated internal testing dataset, with precision, recall, and confusion-matrix performance evaluated as secondary measures. Separately, the study assessed the technical feasibility of developing a high-performing image-classification model using a commercially available, code-free cloud platform without custom model programming or dedicated local high-performance computing resources. Accordingly, the reported results represent image-level internal benchmark performance and technical feasibility rather than clinical diagnostic validation.

## Materials and methods

Dataset and study design

The model was developed to classify individual pediatric chest radiographs into one of two mutually exclusive categories: Normal or Pneumonia. All images contained within the source dataset’s pneumonia category, including bacterial and viral pneumonia subclasses where represented, were combined into a single Pneumonia class. Images from the Normal folder constituted the Normal class, and no etiologic subclassification of pneumonia was performed.

Chest radiographs were obtained from the publicly available chest X-ray images (pneumonia) dataset distributed through Kaggle [17]. The dataset was derived from the pediatric chest radiograph collection originally described by Kermany et al. [4]. The source archive contained 5,863 JPEG images organized into predefined training, validation, and testing directories, each containing Normal and Pneumonia folders. For the present binary classification task, all images contained within the Pneumonia folders were assigned to a single Pneumonia class, including bacterial and viral pneumonia subtypes where represented in the source dataset. Images contained within the Normal folders were assigned to the Normal class. Bacterial and viral pneumonia were not analyzed as separate etiologic categories. The images consisted of anterior-posterior chest radiographs obtained from children approximately one to five years of age at Guangzhou Women and Children’s Medical Center in Guangzhou, China. According to the source publication and dataset description, low-quality or unreadable images were removed, diagnostic labels were reviewed by two expert physicians, and images in the original evaluation set underwent additional review by a third physician [4,17].

The dataset was downloaded as a compressed archive and uploaded to a Google Cloud Storage environment before import into Google Cloud Vertex AI AutoML Image Classification. No manual modification of image content was performed. Prior to model training, the investigators did not conduct a dedicated systematic screen for exact or near-duplicate images, embedded annotations, laterality markers, image borders, or potentially label-associated metadata. Accordingly, the presence of duplicate images or non-pathologic visual cues that could have influenced model predictions cannot be excluded and is acknowledged as a study limitation.

During import, Vertex AI successfully incorporated 5,824 labeled images, including 1,579 normal radiographs and 4,245 pneumonia radiographs. The class labels and original training, validation, or testing assignments of these 39 excluded images could not be reconstructed from the retained import records. Therefore, the excluded images could not be characterized by class or original dataset partition, and exclusion-related selection bias cannot be excluded.

Patient-level identifiers were not available in the publicly distributed dataset. Consequently, the number of unique patients and the presence of multiple images from the same patient could not be determined, and patient-level independence between the training, validation, and testing subsets could not be confirmed. Because repeated or closely related images from the same patient may have been assigned to different subsets, information leakage cannot be excluded and may have contributed to optimistic performance estimates. Accordingly, the reported results should be interpreted strictly as image-level internal benchmark performance rather than patient-independent clinical validation.

Following import, the complete dataset of 5,824 images was pooled within Vertex AI, and the platform-generated random allocation was used for this proof-of-concept analysis. Images were partitioned at the image level into training (n=4,659; 80.0%), validation (n=583; 10.0%), and testing (n=582; 10.0%) subsets. Consequently, the predefined training, validation, and testing partitions from the source dataset were not preserved. Because patient identifiers were unavailable, patient-level separation across the platform-generated subsets could not be verified. This image-level repartitioning may have permitted correlated or repeated images from the same patient to enter different subsets and could therefore have inflated the observed internal performance. Accordingly, the testing results should be interpreted as image-level internal benchmark performance rather than evaluation on an independently preserved held-out test set.

The platform-generated allocation did not preserve the predefined directory-based partitions contained in the downloaded archive. Therefore, the testing subset used in this study represented an internally generated test set and was not equivalent to the original held-out evaluation set described by Kermany et al. [4].

Model development

Model development was performed using Google Cloud Vertex AI AutoML Image Classification (Google LLC, Mountain View, CA, USA) [18]. The classification objective was configured as single-label image classification because each radiograph was assigned to one of two mutually exclusive categories: Normal or Pneumonia.

The model was trained using an eight node-hour budget. All remaining user-configurable training parameters were left at the platform’s default settings. Google Cloud Vertex AI supports cloud-hosted AutoML image-classification training and permits users to specify dataset partitions and a node-hour training budget [18].

No custom neural-network architecture, manually written training code, handcrafted feature extraction, or investigator-defined image-processing pipeline was used. Model architecture selection, training, and optimization were performed automatically by the proprietary AutoML workflow.

Any platform-level image resizing, normalization, augmentation, or hyperparameter optimization was controlled by Vertex AI and was not fully disclosed to the investigators. Model development and training were completed using cloud-based computational resources, eliminating the need for dedicated local graphics processing unit hardware.

After training was completed, the final model was evaluated using the testing subset reserved by the platform. After training was completed, the final model was evaluated using the testing subset reserved by the platform. The trained model was then deployed to a Google Cloud Vertex AI endpoint, which is a hosted online inference service that allows images to be submitted to the deployed model and returns predicted class labels with associated confidence scores. This endpoint was used to generate the representative model predictions shown for selected chest radiographs.

Performance metrics

Model performance was evaluated using metrics generated automatically by Google Cloud Vertex AI. The primary evaluation was performed at a confidence threshold of 0.50, which was the platform-default operating threshold and was not selected on the basis of a prespecified clinical sensitivity or specificity target. Accordingly, the 0.50 threshold should be interpreted as a standardized benchmark operating point rather than a clinically optimized decision threshold. Precision and recall were additionally examined across the range of available confidence thresholds to assess the trade-off between these measures; however, exact class-specific sensitivity and specificity values at alternative thresholds were not retained and therefore could not be reliably reconstructed. Future clinical validation studies should prospectively select operating thresholds according to the intended use case, such as prioritizing sensitivity for screening applications or specificity when minimizing false-positive classifications is more important.

Pneumonia was prespecified as the positive class for class-specific diagnostic-performance calculations. The platform-reported performance measures included average precision, aggregate precision, aggregate recall, a precision-recall curve, precision and recall across confidence thresholds, and a row-normalized confusion matrix. At the 0.50 confidence threshold, the platform reported an overall precision and recall of 98.6%. These values correspond to the overall proportion of correctly classified images (574/582; 98.6%) and were therefore interpreted as micro-averaged aggregate measures rather than Pneumonia-specific precision and recall. Google Cloud documentation identifies overall classification evaluation measures, including area under the precision-recall curve, as micro-averaged at the overall model-evaluation level [18]. Class-specific diagnostic-performance measures were calculated separately from the confusion-matrix counts with Pneumonia designated as the positive class.

The average precision summarized model performance across the precision-recall curve. Precision represented the proportion of positive model predictions that were correct, whereas recall represented the proportion of labeled positive images correctly identified by the model.

The confusion matrix summarized the classification outcomes for each true-label category and was used to derive class-specific diagnostic-performance measures. With Pneumonia designated as the positive class, the testing subset contained 153 true-negative, five false-positive, 421 true-positive, and three false-negative classifications. These counts were used to calculate conventional class-specific diagnostic-performance measures, including sensitivity, specificity, positive predictive value, negative predictive value, F1 score, balanced accuracy, overall accuracy, and corresponding 95% confidence intervals.

Because exact confusion-matrix counts were available, additional diagnostic-performance measures were calculated with Pneumonia designated as the positive class. These measures included overall accuracy, sensitivity, specificity, positive predictive value, negative predictive value, F1 score, and balanced accuracy. Ninety-five percent confidence intervals were estimated using nonparametric bootstrap resampling of the 582 test-set classification outcomes with 10,000 resamples. The analysis was descriptive, and no formal hypothesis testing was performed.

Representative model predictions were generated using the deployed Vertex AI endpoint. Two correctly classified normal radiographs and two correctly classified pneumonia radiographs were selected post hoc to illustrate the model-output format. These examples were not randomly selected and were not used to calculate or validate model performance.

Ethical considerations

Institutional review board approval was not required because the study used only publicly available, de-identified imaging data and did not involve direct interaction with human participants or access to identifiable private information.

No protected health information was accessed. The model was developed and evaluated exclusively for research purposes, and no model predictions were used for clinical decision-making or patient care.

## Results

A total of 5,824 of 5,863 source chest radiographs (99.3%) were successfully imported into Google Cloud Vertex AI, while 39 images (0.7%) were not imported. The imported dataset included 1,579 normal radiographs (27.1%) and 4,245 pneumonia radiographs (72.9%). The platform randomly allocated 4,659 images (80.0%) to training, 583 images (10.0%) to validation, and 582 images (10.0%) to testing. The testing subset contained 158 normal radiographs (27.1%) and 424 pneumonia radiographs (72.9%), closely reflecting the overall dataset distribution. Class-specific counts for the training and validation subsets were not retained in the available Vertex AI records and therefore could not be reported separately. Consequently, preservation of comparable class proportions across those two subsets could not be independently verified.

The platform randomly allocated 4,659 images (80.0%) to the training subset, 583 images (10.0%) to the validation subset, and 582 images (10.0%) to the testing subset. The model was trained using an eight node-hour budget, with an actual training time of two hours and eight minutes. A summary of the dataset characteristics, model-training allocation, and performance metrics is shown in Table 1.

**Table 1:** Dataset characteristics and model performance summary. This table summarizes the number of source and successfully imported chest radiographs, the distribution of images across the training, validation, and testing subsets, the model-training budget and duration, and the principal performance metrics reported by Google Cloud Vertex AI. The confusion-matrix values represent the percentage of radiographs correctly classified within each true-label category. Exact class-specific counts for the platform-generated subsets and exact confusion-matrix cell counts were not available in the retained output.

Metric	Value
Source images	5,863
Successfully imported images	5,824
Images not imported	39
Normal images	1,579
Pneumonia images	4,245
Training images	4,659
Validation images	583
Testing images	582
Training budget	Eight node-hours
Actual training time	Two hours, eight minutes
Average precision	0.999
Precision at threshold 0.50	98.60%
Recall at threshold 0.50	98.60%
Normal images correctly classified	97%
Pneumonia images correctly classified	99%

**Figure 1:**
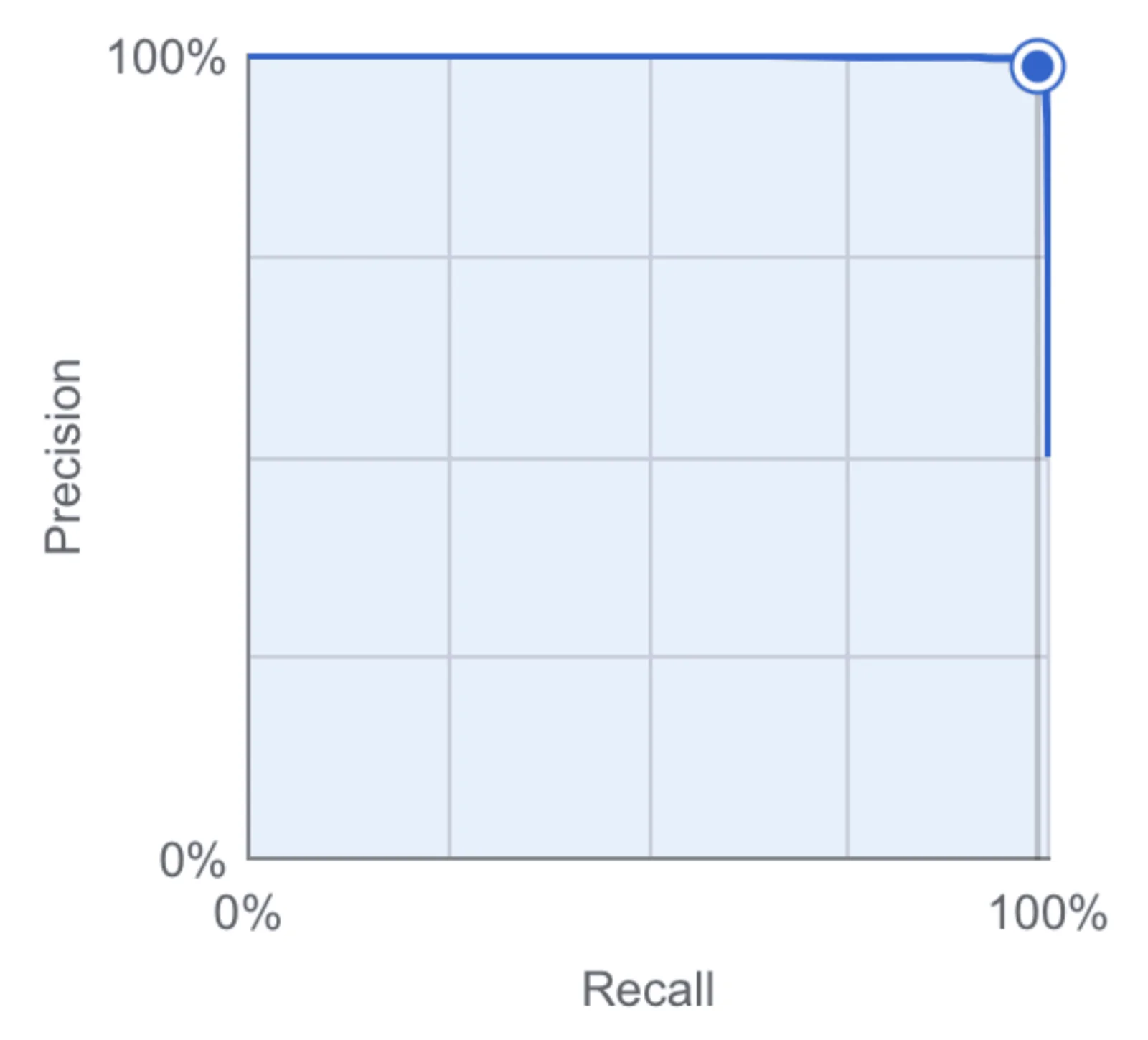
Precision-recall curve for the pediatric pneumonia image-classification model. The precision-recall curve generated by Google Cloud Vertex AI demonstrates consistently high precision across a broad range of recall values. The model achieved an average precision of 0.999, indicating strong discrimination between normal and pneumonia chest radiographs within the internal testing dataset.

Upon evaluation of the internal testing subset, the model achieved an average precision of 0.999. The precision-recall curve remained near the upper-right region of the graph across most recall values, demonstrating strong discrimination between normal and pneumonia chest radiographs (Figure 1).

**Figure 2:**
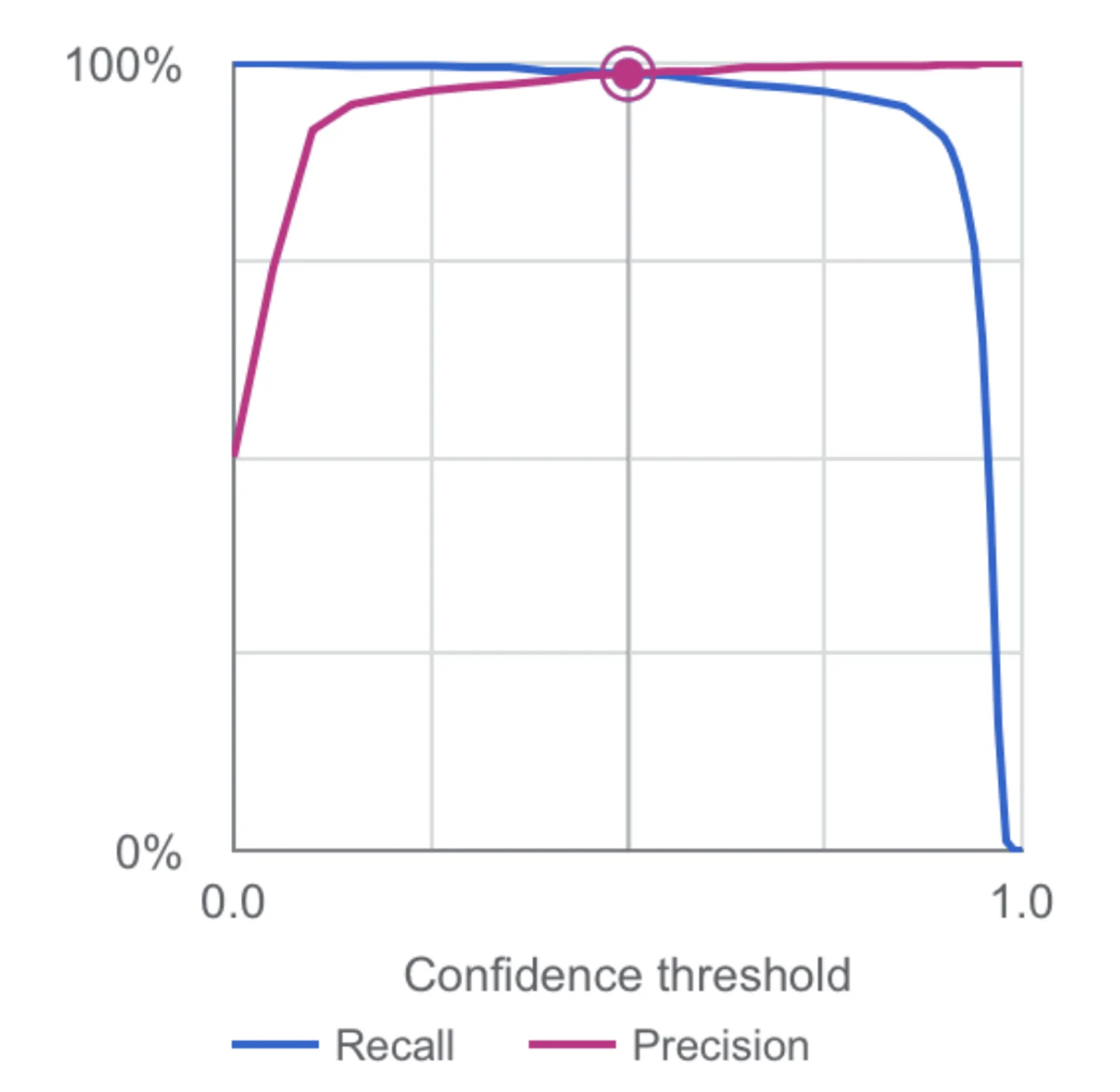
Precision and recall across varying confidence thresholds. This plot illustrates changes in model precision and recall across different confidence thresholds. Both metrics remained high across most of the evaluated range. The primary analysis was performed at the default confidence threshold of 0.50, at which precision and recall were both 98.6%.

At the default confidence threshold of 0.50, the platform-reported aggregate precision and recall were both 98.6%. In this single-label binary classification setting, these aggregate values corresponded numerically to the overall accuracy of 574 correctly classified images among 582 test images (98.6%) and were interpreted as micro-averaged measures rather than Pneumonia-specific precision and recall. With Pneumonia designated as the positive class, sensitivity was 99.3% (421/424), specificity was 96.8% (153/158), positive predictive value was 98.8% (421/426), and negative predictive value was 98.1% (153/156). Precision and recall remained high across most evaluated confidence thresholds, with recall declining at higher thresholds while precision remained comparatively stable. Precision and recall remained high across most evaluated confidence thresholds. Recall declined as the confidence threshold approached 1.0, while precision remained comparatively stable (Figure 2).

**Figure 3:**
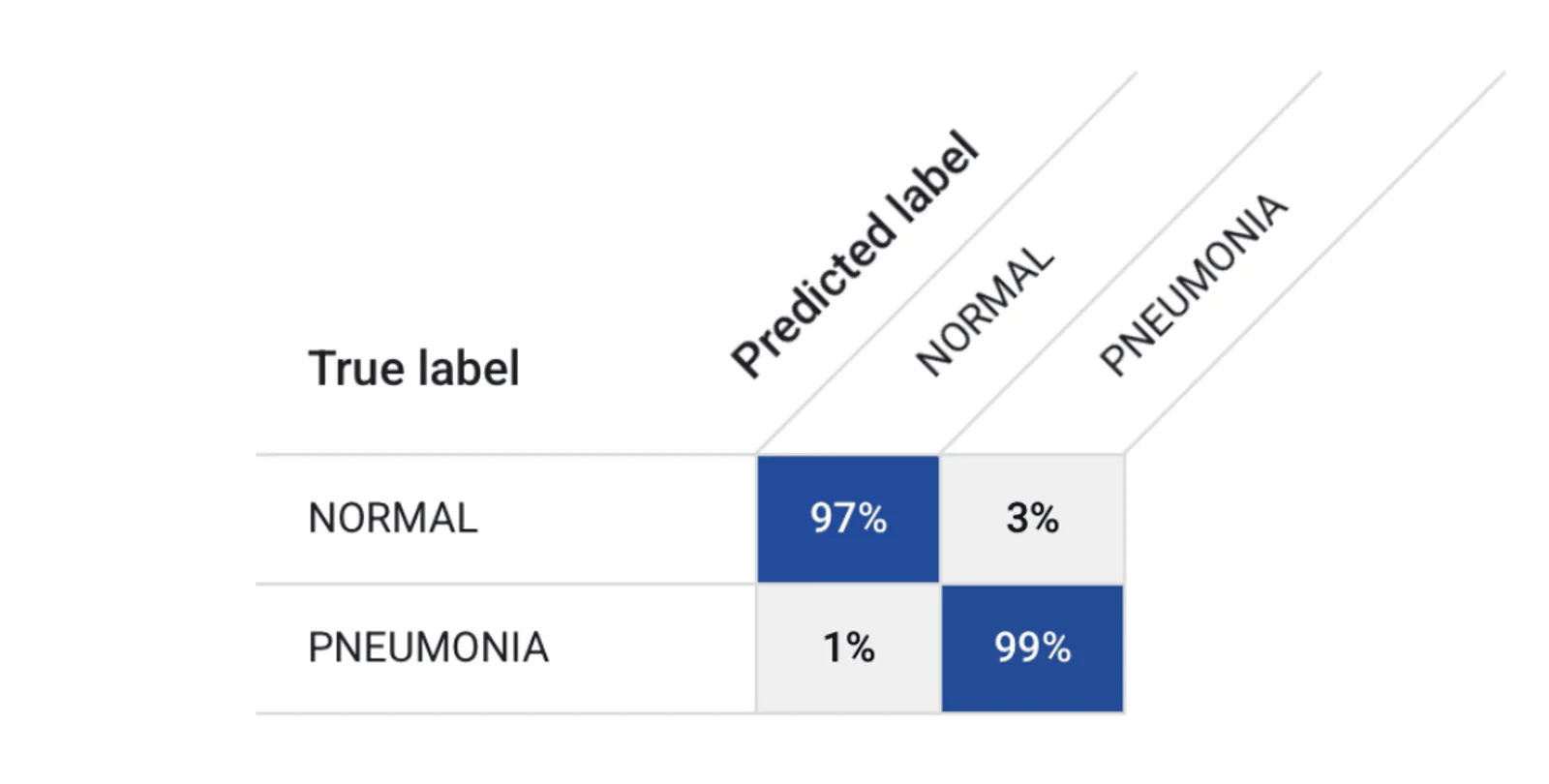
Confusion matrix for the pneumonia image-classification model. Rows represent the true image labels, and columns represent the model-predicted labels. Among 158 normal radiographs, 153 (96.8%) were correctly classified as Normal and five (3.2%) were classified as Pneumonia. Among 424 pneumonia radiographs, 421 (99.3%) were correctly classified as Pneumonia and three (0.7%) were classified as Normal.

Performance was further evaluated using a row-normalized confusion matrix. Among 158 radiographs with a true label of Normal, 153 were correctly classified as Normal, corresponding to a specificity of 96.8% (95% CI, 92.8%-98.6%), while five were classified as Pneumonia, corresponding to a false-positive rate of 3.2% (95% CI, 1.4%-7.2%). Among 424 radiographs with a true label of Pneumonia, 421 were correctly classified as Pneumonia, corresponding to a sensitivity of 99.3% (95% CI, 97.9%-99.8%), while three were classified as Normal, corresponding to a false-negative rate of 0.7% (95% CI, 0.2%-2.1%) (Figure 3).

**Figure 4:**
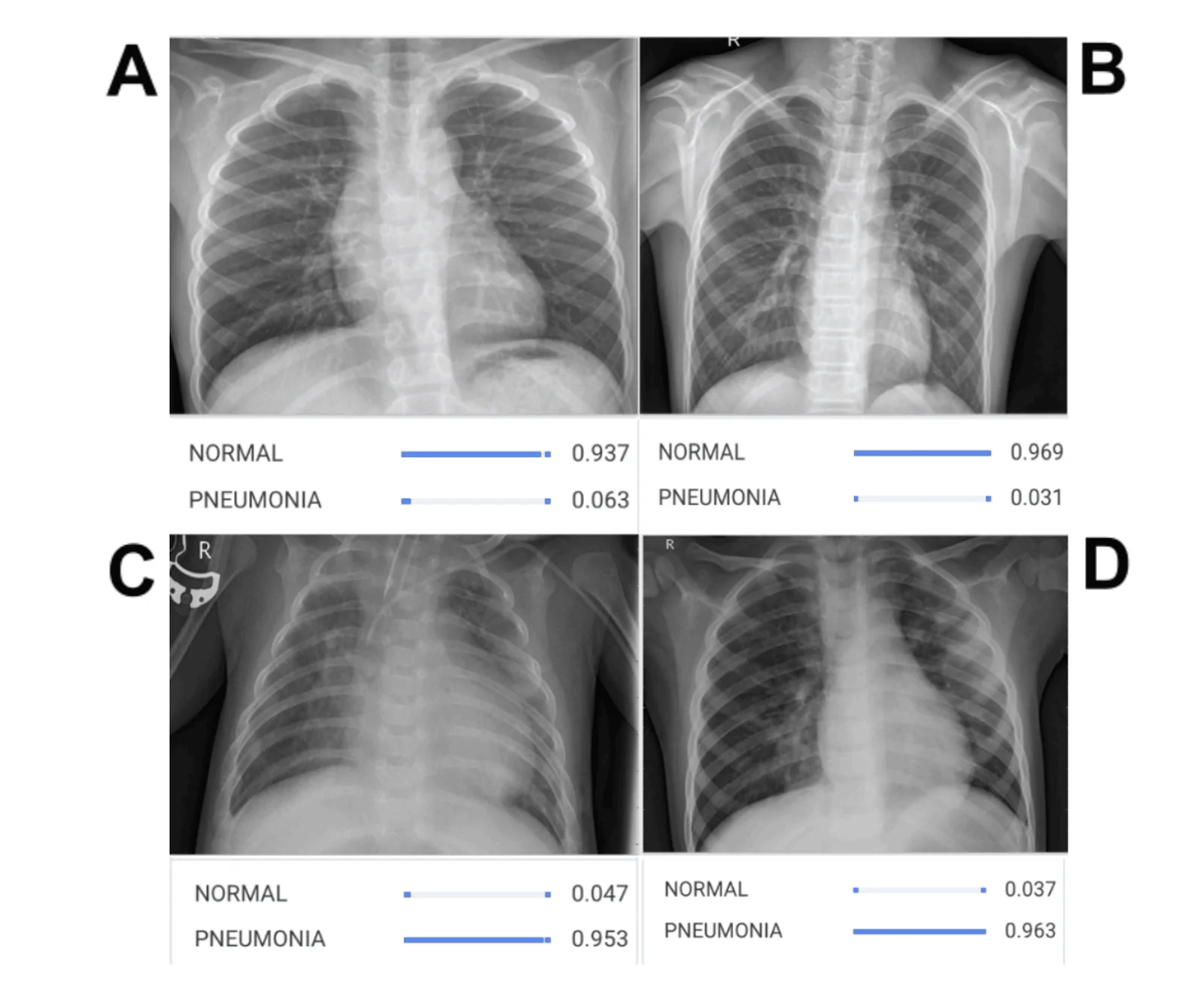
Representative predictions generated by the pneumonia image-classification model. Panels A and B show chest radiographs correctly classified as Normal, with predicted Normal probabilities of 0.937 and 0.969, respectively. Panels C and D show radiographs correctly classified as Pneumonia, with predicted Pneumonia probabilities of 0.953 and 0.963, respectively. The horizontal bars beneath each radiograph display the probabilities assigned to the Normal and Pneumonia classes. These images were selected to illustrate the model-output format.

To illustrate the model’s prediction format, two correctly classified normal radiographs and two correctly classified pneumonia radiographs were selected as representative examples. The two normal radiographs received predicted Normal probabilities of 0.937 and 0.969. The two pneumonia radiographs received predicted Pneumonia probabilities of 0.953 and 0.963 (Figure 4).

The four representative radiographs were selected post hoc from correctly classified cases to illustrate the model-output format, with two Normal and two Pneumonia examples chosen to demonstrate high-confidence predictions from each class. The examples were not randomly selected and were not used in calculating model performance; therefore, they should be interpreted as illustrative rather than representative of the overall testing distribution.

## Discussion

This study demonstrated that Google Cloud Vertex AI AutoML Image Classification could develop a high-performing binary classifier for pediatric chest radiographs without requiring custom programming, manual neural-network design, or dedicated local high-performance computing infrastructure. The model achieved an average precision of 0.999 and platform-reported precision and recall of 98.6% on the internally generated testing subset.

The performance observed in this study is broadly consistent with previous investigations reporting strong results for deep-learning approaches to pneumonia classification on chest radiographs [2-7]. However, direct numerical comparisons should be interpreted cautiously because prior studies differ in dataset partitioning, patient-level separation, test-set composition, preprocessing, model architecture, reference standards, and validation methodology. Kermany et al. established the feasibility of deep-learning-based pneumonia classification using the pediatric dataset from which the present images were derived [4]. Subsequent systematic reviews and meta-analyses have likewise reported high performance across multiple convolutional neural-network approaches, although substantial methodological heterogeneity limits direct comparison with the present results [3,5-7]. Deep-learning systems have also demonstrated performance comparable to practicing clinicians for selected chest radiograph interpretation tasks [8]; however, those findings were obtained using different datasets and validation frameworks and should not be interpreted as directly equivalent to the image-level internal benchmark performance reported in this study.

The principal contribution of the present study is not the development of a novel neural-network architecture, but rather the application of a commercially available, code-free AutoML workflow specifically to pediatric pneumonia classification on chest radiographs using a widely recognized benchmark dataset. Prior code-free medical image-classification studies have demonstrated the general feasibility of AutoML across heterogeneous imaging tasks and modalities [10-12]. The present study extends that work by evaluating a pediatric chest radiograph use case, documenting an end-to-end Google Cloud Vertex AI workflow, and quantifying image-level internal benchmark performance using class-specific diagnostic metrics derived from the confusion matrix. In addition, the study highlights practical tradeoffs that are less apparent in architecture-focused work, including reduced programming and local hardware requirements alongside limitations related to proprietary model development, vendor dependence, cloud costs, data governance, and reproducibility. Thus, the incremental contribution is primarily methodological and translational rather than architectural.

The model demonstrated strong classification performance for both normal and pneumonia radiographs. However, the retained platform output initially displayed rounded percentages rather than complete prediction-level results. Exact confusion-matrix counts are necessary for transparent reporting and for independently calculating confidence intervals, specificity, predictive values, F1 score, and overall accuracy. Complete image-level prediction outputs were not retained from the present Vertex AI evaluation and could not be recovered for reanalysis. This prevented a complete case-level audit beyond the available confusion-matrix counts and represents an important limitation of the current study. Accordingly, the present findings should be interpreted as aggregate image-level internal benchmark performance, and future studies should prospectively retain prediction-level outputs to enable full statistical, error, calibration, and subgroup analyses.

Representative predictions demonstrated that the deployed model could assign high confidence to selected normal and pneumonia radiographs. However, these images were selected post hoc from correctly classified examples and were included only to demonstrate the model-output format. They should not be interpreted as representative of the entire testing dataset or as evidence that the model consistently identified the pulmonary abnormalities responsible for the assigned labels. A structured review of all eight misclassified cases would have strengthened the analysis; however, the individual false-positive and false-negative image-level outputs were not retained and could not be recovered from the available Vertex AI records. Consequently, a case-level error analysis could not be performed in the present study. This limitation restricts assessment of whether misclassifications were associated with subtle pulmonary findings, image quality, positioning, annotations, or other dataset-specific features and should be considered when interpreting the reported performance.

Despite the strong internal performance, these findings should not be interpreted as evidence of clinical readiness. The model was developed and evaluated using a single curated pediatric dataset from one institution. The dataset contained only two categories and did not represent the full range of findings encountered in routine chest radiography. In clinical practice, pneumonia may coexist with or resemble atelectasis, pulmonary edema, aspiration, viral infection, chronic lung disease, congenital abnormalities, postoperative changes, indwelling devices, and technical artifacts.

Artificial intelligence models may learn dataset-specific signals rather than clinically meaningful disease features. In the present study, the complete dataset was not systematically screened before training for exact or near-duplicate images, embedded annotations, laterality markers, image borders, positioning differences, or other potentially label-associated non-pathological cues. In addition, the individual outputs for the eight misclassified cases were not retained and could not be recovered, preventing a structured retrospective error analysis. Saliency or other explainability analyses were also not performed because prediction-level outputs and model-internal information required for such analyses were not available from the retained proprietary AutoML workflow. Consequently, the possibility that model predictions were influenced by duplicated images or dataset-specific artifacts rather than pulmonary findings cannot be excluded. Differences in image acquisition, resolution, processing, equipment, or institutional workflow may enable a model to exploit non-pathological source-specific features rather than the underlying disease process. Zech et al. demonstrated that the performance of a pneumonia-detection model varied substantially across hospital systems, highlighting the risks of hidden institutional bias and limited external generalizability [14]. Independent validation using radiographs from different institutions and patient populations is therefore essential before clinical application.

The proprietary nature of Vertex AI presents additional challenges for transparency and reproducibility. The platform automated architecture selection, preprocessing, augmentation, training, and optimization, but the complete procedures were not disclosed to the investigators. Commercial platforms and their default settings may also change over time. Although this automation increases accessibility, it limits the ability of independent researchers to reproduce the complete modeling workflow or determine which imaging features most strongly influenced predictions. 

Future investigations should preserve patient-level separation between training, validation, and testing datasets and retain exact image-level predictions. External validation should be performed using independent multi-institutional datasets representing different imaging systems, acquisition protocols, geographic regions, and clinical settings. Additional research should include calibration analysis, subgroup evaluation, systematic review of misclassified images, comparison with radiologists and established open-source models, and prospective workflow assessment.

Limitations

This study has several important limitations. First, model development and evaluation were performed using a single publicly available pediatric dataset obtained from one institution, limiting generalizability to other institutions, adult populations, and real-world clinical settings. Second, Vertex AI randomly reassigned the imported images, and patient identifiers were unavailable; therefore, patient-level separation between the training, validation, and testing subsets could not be verified. Third, the dataset was imbalanced and included only the binary labels Normal and Pneumonia, which do not reflect the broader spectrum of chest radiograph abnormalities encountered in clinical practice. Fourth, the proprietary AutoML workflow did not fully disclose the model architecture, preprocessing, augmentation, or optimization procedures. Finally, no external validation, calibration analysis, subgroup analysis, radiologist comparison, or prospective clinical evaluation was performed. Finally, no external validation, calibration analysis, subgroup analysis, radiologist comparison, prospective clinical evaluation, or comparison with an open-source baseline model trained and tested using the same data partitions was performed. The absence of such a baseline limits the ability to determine whether the observed performance reflects an advantage of the AutoML workflow itself versus performance achievable with a conventional open-source model under identical experimental conditions.

Although cloud-based AutoML may reduce requirements for local computing infrastructure and specialized programming expertise, accessibility remains dependent on reliable internet access, cloud-computing costs, institutional data-governance requirements, and reliance on a commercial vendor. Vendor-specific pricing, platform availability, proprietary model-development processes, and changes to cloud services over time may also affect reproducibility and implementation. These considerations should be weighed alongside the technical accessibility of code-free model development.

## Conclusions

Google Cloud Vertex AI AutoML Image Classification achieved strong image-level internal benchmark performance for binary classification of pediatric chest radiographs as Normal or Pneumonia, with an average precision of 0.999 and overall accuracy of 98.6%. The model was developed without custom programming or dedicated local high-performance computing resources, demonstrating the technical feasibility of a code-free cloud-based AutoML workflow for pediatric chest radiograph classification.
